# Survival benefit of combined immunotherapy and chemoradiotherapy in locally advanced unresectable esophageal cancer: an analysis based on the SEER database

**DOI:** 10.3389/fimmu.2024.1334992

**Published:** 2024-01-16

**Authors:** Liangyun Xie, Zhi Zhang

**Affiliations:** ^1^ Hebei Medical University, Shijiazhuang, China; ^2^ Department of Radiation Oncology, Affiliated Tangshan Worker’s Hospital, Hebei Medical University, Tangshan, China

**Keywords:** esophageal cancer, SEER, unresectable, chemoradiotherapy, immunotherapy, survival analysis, prognosis

## Abstract

**Background:**

While simultaneous chemoradiotherapy remains the established therapeutic modality for patients afflicted with locally advanced esophageal cancer, the effectiveness of this radical approach falls short of the desired outcome. Numerous investigations have illuminated the prospect of enhancing therapeutic efficacy through the amalgamation of chemoradiotherapy and immunotherapeutic interventions. Consequently, we embarked on an examination to scrutinize the potential survival advantages conferred by the confluence of chemoradiotherapy and immunotherapy in relation to locally advanced unresectable esophageal carcinoma, drawing upon the extensive SEER database for our analysis.

**Methods:**

We extracted clinicopathological attributes and survival statistics of patients afflicted with locally advanced unresectable esophageal carcinoma, diagnosed within the temporal span encompassing the years 2004-2014 and 2019-2020, from the extensive SEER database. To discern disparities in both overall survival (OS) and cancer-specific survival (CSS) between the cohorts subjected to chemoradiotherapy combined with immunotherapy and chemoradiotherapy alone, we employed analytical tools such as Kaplan-Meier analysis, the Log-rank test, the Cox regression proportional risk model, and propensity-matched score (PSM) methodology.

**Results:**

A total of 7,758 eligible patients were encompassed in this research, with 6,395 individuals having undergone chemoradiotherapy alone, while 1,363 patients received the combined treatment of chemoradiotherapy and immunotherapy. After 1:4 propensity score matching, 6,447 patients were successfully harmonized, yielding a well-balanced cohort. The Kaplan-Meier curves demonstrated a substantial enhancement in OS (P = 0.0091) and CSS (P < 0.001) for the group subjected to chemoradiotherapy combined with immunotherapy as compared to chemoradiotherapy alone. Further multivariable analysis with PSM confirmed that chemoradiotherapy combined with immunotherapy benefits OS(HR=0.89, 95% CI 0.81-0.98) and CSS (HR=0.68, 95% CI 0.61-0.76). In addition, Univariable and multivariable Cox regression analyses of the matched patient groups unveiled several independent prognostic factors for OS and CSS, including sex, age, marital status, tumor location, tumor size, pathologic grade, SEER historic staging, and treatment modality. Among these factors, being female, married, and receiving chemoradiotherapy combined with immunotherapy emerged as independent protective factors, while age exceeding 75 years, non-superior segment tumor location, tumor size greater than 6 cm, Grade 3-4 pathology, and regional SEER historic staging were all found to be independent risk factors. The survival advantage of the chemoradiotherapy combined with the immunotherapy group over the chemoradiotherapy alone group was substantial.

**Conclusions:**

This investigation furnishes compelling evidence that the integration of immunotherapy with chemoradiotherapy confers a noteworthy survival advantage when contrasted with conventional chemoradiotherapy for individuals grappling with locally advanced unresectable esophageal carcinoma.

## 1 Introduction

Esophageal cancer stands as the seventh most prevalent malignancy globally, ranking as the sixth primary reason for cancer-related fatalities. According to the 2020 global cancer statistics, esophageal cancer accounts for one out of every 18 cancer-related deaths. A striking 70% of esophageal cancer diagnoses affect males, with an incidence and mortality rate two to three times higher in men compared to women (1). There are two predominant pathological subtypes of esophageal cancer: squamous carcinoma and adenocarcinoma. In Western regions, adenocarcinoma prevails, while in East Asia and the Middle East, squamous carcinoma constitutes the majority, representing approximately 90% of all cases (2). The preferred treatment for early-detected esophageal cancer with superficial lesions is surgical intervention. However, early clinical manifestations of esophageal cancer often manifest atypically, eluding patient detection. Consequently, the majority of esophageal cancer cases are diagnosed at an intermediate to advanced stage, precluding the option of surgery (3). For these patients, the primary treatment approach involves chemoradiotherapy-based combination therapy, yet the prognosis remains unfavorable, with a mere 20% 5-year survival rate (4–6). Furthermore, over 50% of locally advanced patients face disease recurrence or progression following chemotherapy, and their median survival spans a mere 4 to 28 months (7).

In recent years, the landscape of esophageal cancer treatment has evolved substantially with the rapid advancement of immunotherapy. Immunotherapy, when combined with chemotherapy, has emerged as the first-line treatment for advanced esophageal cancer, delivering promising disease control outcomes. Radiotherapy, as a pivotal component of esophageal cancer management, is believed to possess synergistic potential when combined with immunotherapy, and investigations into the efficacy of this approach are ongoing (8). A small-scale study enlisted 20 cases of locally advanced, inoperable esophageal cancer patients. Post-receipt of concurrent chemoradiotherapy coupled with immunotherapy, all subjects underwent immune maintenance therapy after the completion of radiotherapy. The study findings reveal that the overall survival rates for this cohort at 12 and 24 months stood at 85.0% and 69.6%, respectively. Progression-free survival rates were documented at 80.0% and 65.0%, correspondingly. Noteworthy grade 3 treatment-related adverse events included radiation esophagitis (20%) and esophageal fistula (10%). Eight cases (40%) encountered severe treatment-related adverse events, mirroring rates reported in preceding studies. This diminutive yet pioneering study signifies a landmark effort in amalgamating immunotherapy with concurrent chemoradiotherapy for locally advanced esophageal cancer, attaining a novel pinnacle in treatment efficacy for such cases. It establishes a crucial foundation for prospective research endeavors in this domain (9). Currently, five randomized phase III clinical trials (ESCORT-CRT, KEYNOTE-975, RATIONALE-311, KUNLUN, and SKYSCRAPER-07) are underway for unresectable locally advanced esophageal cancer. However, the existing body of evidence is limited and warrants further exploration. Thus, additional studies investigating the combination of chemoradiotherapy and immunotherapy in patients with locally advanced esophageal cancer are imperative to bolster this perspective.

The National Cancer Institute’s (NCI) Surveillance, Epidemiology, and End Results (SEER) database serves as a comprehensive national cancer repository, meticulously documenting demographic and clinical information for nearly one-third of the United States population. It stands as a definitive source of data concerning cancer incidence and survival in the United States. In the nascent stages of the SEER database’s inception, there existed a mere nine initial tumor registries. Now, the project has burgeoned to encompass twenty-two geographically diverse areas across the United States, enveloping approximately 48% of the nation’s entire population of cancer patients. The overarching objective of this research is to leverage the extensive dataset of the SEER database to assess whether the amalgamation of chemoradiotherapy and immunotherapy bestows a notable survival advantage upon individuals grappling with locally advanced unresectable esophageal carcinoma.

Considering the prevalent incidence of locally advanced unresectable esophageal cancer, its relatively poor prognosis, and the limited extent of research on immunotherapy for such patients, we conducted an analysis using the Surveillance, Epidemiology, and End Results (SEER) database. The aim was to comprehend the impact of combined chemoradiotherapy and immunotherapy in this specific patient population. Moreover, the focus of this study was on the role of chemoradiotherapy combined with immunotherapy for OS and CSS in patients. We further explored significant prognostic factors affecting this patient population, thereby offering additional evidence for decision-making in clinical practice and providing more reference data for the design of future clinical trials.

## 2 Materials and methods

### 2.1 Data sources and patient selection

This study is predicated on the November 2022 release of the SEER database. All patients were sourced from SEER*Stat version 8.4.2, encompassing population data related to cancer across 17 cancer registries from 2000 to 2020. This version of the database, covering approximately 30% of the U.S. population, provides comprehensive information concerning patient demographics, tumor characteristics, diagnoses, initial treatment regimens, and vital status updates. It is noteworthy that the SEER database upholds strict patient confidentiality and does not reveal personally identifiable information. Therefore, data analysis for this study was exempt from medical ethical review and did not necessitate the acquisition of informed consent from participants. All procedures undertaken in this study involving human subjects adhered to the guidelines established by the Declaration of Helsinki, which was published in 1964, and its subsequent amendments or equivalent ethical standards. Based on the third edition of the International Classification of Diseases of Oncology (ICD-O-3) and the SEER historical staging system, we conducted a comprehensive screening of patients suffering from locally advanced esophageal carcinoma, spanning the diagnosis years from 2000 to 2020. In 2019, the U.S. Food and Drug Administration (FDA) granted approval to K-drug (pembrolizumab) for the treatment of advanced esophageal cancer, marking the pioneering PD-1 immunotherapeutic drug sanctioned for esophageal cancer treatment. The specific indication pertains to PD-L1-positive recurrent locally advanced or metastatic esophageal squamous cell carcinoma. This momentous achievement dismantles the longstanding impasse in esophageal cancer treatment that endured for nearly half a century, conclusively demonstrating the superior efficacy of K-drug monotherapy over conventional chemotherapy. This approval not only signifies the resolution of a decades-long deadlock but also heralds the official entry of esophageal cancer into the era of immunization. To evaluate the effects of immunotherapy, we specifically targeted patients diagnosed with esophageal cancer in the years 2019-2020, aligning with the approval of immunotherapy as a primary treatment modality in 2019. Patients diagnosed between 2004 and 2014 served as the comparative cohort for this analysis. Initially, patients diagnosed with cancer localized in the esophagus were selected based on the International Classification of Diseases of Oncology, Third Edition (ICD-O-3) part codes (C15.1-C15.9) corresponding to the primary site, while individuals with other malignancies were systematically excluded. The diagnostic timeframe was defined as 2004-2014 and 2019-2020. Subsequently, demographic and clinical data were meticulously collected, encompassing sex, age, race, marital status, tumor location, tumor size, pathologic grade, pathologic type, SEER historical staging system, SEER-documented primary cause of death, survival time, survival status, radiation therapy, chemotherapy, and the year of initial diagnosis. The code of “Dead” (attributable to this cancer dx) was used to identify deaths due to esophageal cancer, whereas “other codes for death” (dead of other cause and N/A not first tumor) defined mortality from other causes. The SEER staging system encompasses four distinct staging categories: ‘in situ,’ ‘local,’ ‘regional,’ and ‘distant disease.’ For the purposes of this study, ‘local disease’ was defined as a tumor restricted to a localized anatomical region without breaching the esophageal mucosal membrane and devoid of regional lymph node involvement (T1-2N0M0). ‘Regional disease’ was characterized as a tumor confined to the regional anatomical area with no signs of distant metastasis (T3-4aN0M0/T1-4aN1-3M0).

**Figure 1:**
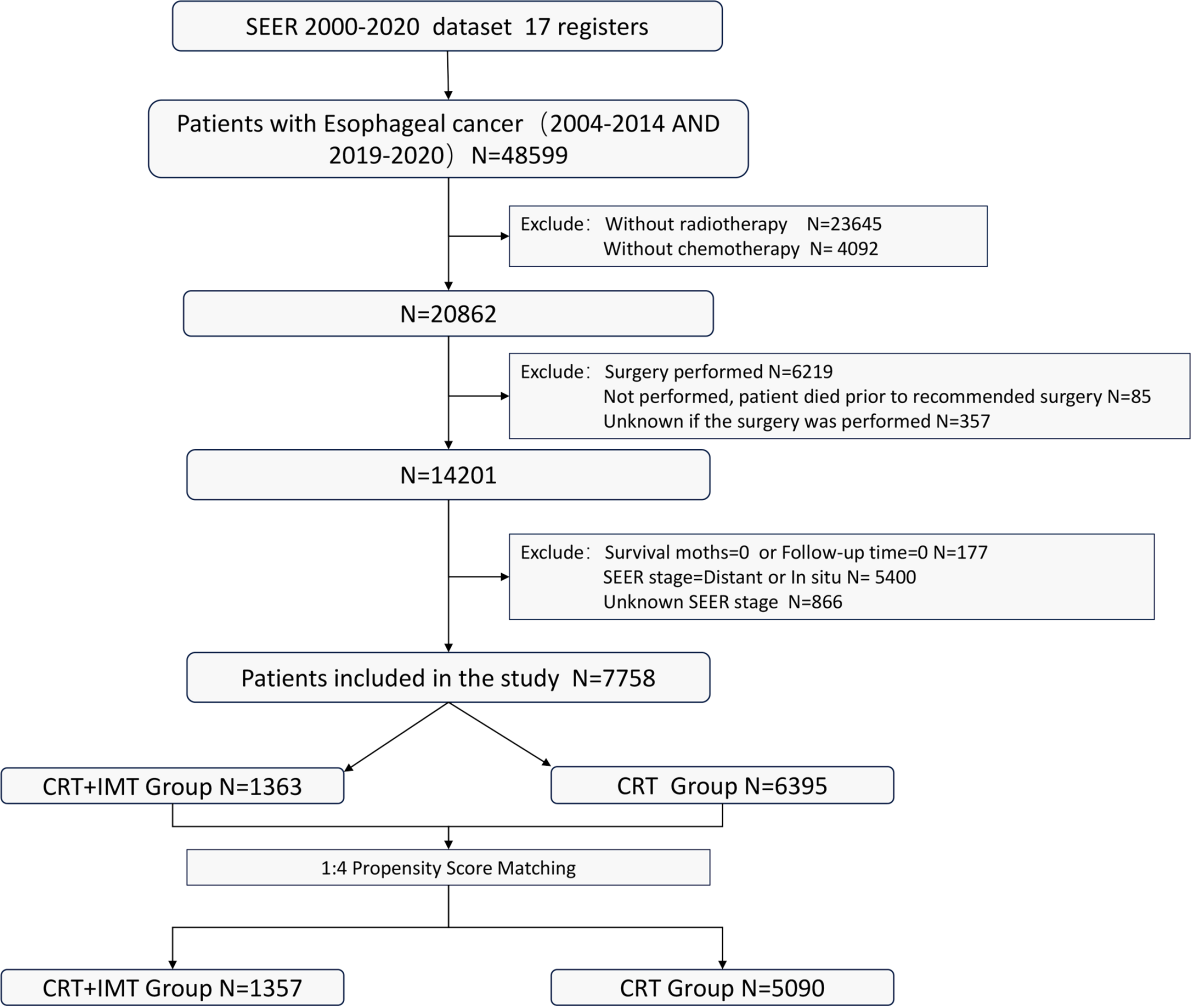
Flowchart of the patients screening process. CRT, chemoradiotherapy; IMT, immunotherapy.

Inclusion criteria: (I) Patients who received a combined regimen of radiation therapy and chemotherapy; (II) Patients for whom surgical intervention was either not advised or declined by the patient; (III) Individuals aged 18 years or older. Exclusion criteria: (I) Patients who had undergone surgical procedures, those for whom surgical information remained unrecorded, or individuals who passed away prior to the recommended surgical intervention; (II) Cases with insufficient or incomplete treatment and follow-up data; Patients with a survival duration of 0 days; (III) Individuals diagnosed with carcinoma in situ, those who exhibited distant metastasis, or those with unknown metastatic status. The flowchart illustrating the selection of the research population is depicted in **Figure 1**.

Furthermore, we conducted a retrospective analysis that encompassed 63 patients suffering from locally advanced inoperable ESCC who were treated at our institution between 2019 and 2023. All individuals included in this analysis underwent a combined regimen of chemoradiotherapy and immunotherapy.

### 2.2 Study outcomes

The locally advanced inoperable carcinoma of the esophagus cases sourced from the SEER database were categorized into two distinct groups for analysis. These groups were defined based on the timing of immunotherapy approval as a primary treatment modality for esophageal cancer, resulting in a division between the ‘chemoradiotherapy group’(CRT) and the ‘chemoradiotherapy combined with immunotherapy group’(CRT+IMT).

The primary endpoints of this research encompassed both OS and CSS. OS was characterized as the duration from the point of diagnosis to the occurrence of death due to any cause or until the last available follow-up. On the other hand, CSS was defined as the span from diagnosis to death attributed to esophageal cancer, or, in cases where patients neither succumbed to the disease nor to any cancer-related cause, it extended up to the last documented follow-up. The SEER Cause of Death Classification was utilized to ascertain and document the precise cause of death for each patient. The survival status, as indicated in the SEER database, is denoted as ‘Vital Status,’ while the duration of survival is recorded under ‘Survival months.’

### 2.3 Statistical analysis

The χ2 test was deployed to scrutinize the fundamental clinical characteristics of patients within the ‘CRT+IMT’ group and the ‘CRT’ group. We employed a 23-month cutoff value as the follow-up time for this analysis. Prognostic determinants of esophageal cancer were scrutinized through univariable and multivariable Cox regression analyses, wherein variables exhibiting p-values less than 0.1 in univariable analysis were included in the multivariable analysis. The Kaplan-Meier method was applied to generate OS and CSS curves both before and after propensity score matching within the ‘CRT+IMT’ and ‘CRT’ groups. To assess the statistical significance of the survival outcomes, we applied the log-rank test. Inevitably, selection bias permeated this retrospective investigation owing to the incongruous nature of baseline characteristics. In order to mitigate the prognostic ramifications stemming from dissimilarities in baseline characteristics, a 1:4 propensity score matching (PSM) technique, employing a caliper width of 0.1, was implemented to harmonize patients between the cohorts undergoing chemoradiotherapy in conjunction with immunotherapy and those undergoing chemoradiotherapy alone. Subsequent subgroup analyses were undertaken to measure the resilience of the relationships within treatment subgroups and to explore potential interactions between treatment and other variables. All statistical tests were two-tailed, and statistical significance was established for p-values less than 0.05. The analyses were executed with R 4.2.1 (R Foundation for Statistical Computing, Vienna, Austria). Propensity score matching (PSM) was accomplished utilizing the MatchIt package within R software 4.2.1. Optimal threshold values for age and lesion length were ascertained via X-tile software (Yale University, New Haven, CT, USA). The X-tile software functions as an objective methodology to ascertain the optimal truncation threshold and has found widespread application across various academic inquiries. The stratagem employed by the X-tile software encompasses the evaluation of every numerical datum within the spectrum of a variable as a potential truncation threshold. Following this, the χ2 value and P-value are derived through the utilization of these scrutinized numerical values as truncation thresholds. Ultimately, the numerical value corresponding to the pinnacle χ2 value and the nadir P-value is singled out as the optimum truncation threshold for the specific variable under consideration.

## 3 Results

### 3.1 Selection of study cohort and propensity score matching

**Figure 2:**
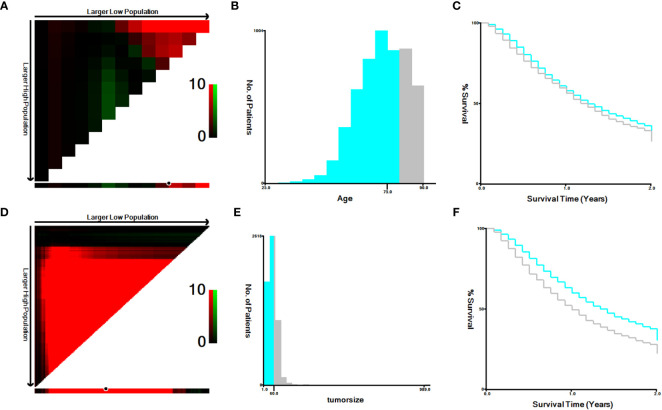
**(A–F)** The image shows defining the optimal cutoff values of age and tumor size via X-tile analysis. **(A, D)** The black dot indicates that optimal cutoff values of age/tumor size have been identified. **(B, E)** A histogram and **(C, F)** Kaplan-Meier curve were constructed based on the identified cutoff values. Optimal cutoff values of age were identified as 75 years based on survival (χ2 = 7.4869, P=0.00617). Optimal cutoff values of tumor size were identified as 60 mm based on survival (χ2 = 69.9293, P<0.0001).

**Figure 3:**
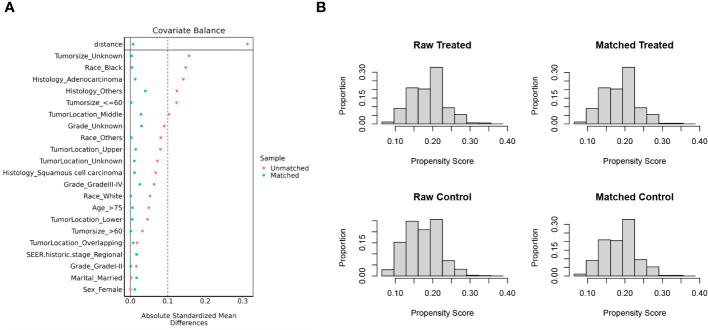
**(A, B)** Propensity score matching between the CRT+IMT group and CRT group described using standardized mean difference (SMD) **(A)** and propensity score **(B)**.

A total of 7,758 patients who had received diagnoses of locally advanced unresectable esophageal cancer were extracted from the SEER database. Among them, 1,363 cases fell under the umbrella of the ‘CRT+IMT’ group, while the remaining 6,395 cases constituted the ‘CRT’ group. X-plots were employed to delineate the optimal threshold values for age and tumor size, culminating in determinations of 75 years and 60 mm, as depicted in **Figure 2**. These optimal cut-off values were used as benchmarks to stratify patients into groups based on age and tumor size. In the cohort of patients with esophageal cancer, males predominated, comprising 74.9% of the cases, with a male-to-female ratio of 2.98:1. Moreover, a significant majority of patients were of Caucasian ethnicity, representing 80.9% of the total. Distinctions of notable significance between the two groups encompass race, tumor location, tumor size, grade classification, and pathology type. To mitigate the influence of confounding variables, a 1:4 propensity score matching (PSM) strategy was employed, resulting in the creation of a final matched cohort comprising 1,357 cases in the ‘CRT+IMT’ group and 5,090 cases in the ‘CRT’ group. Following this matching process, the two cohorts exhibited a high degree of alignment in baseline characteristics, as detailed in **Table 1**. The standardized mean differences (SMDs) between the matched cohorts were consistently below 0.1, and the distribution of scores in both groups displayed a remarkable degree of uniformity, as illustrated in **Figure 3**.

**Table 1:** Baseline characteristics of patients before and after propensity score matching.

Variables	Original data set(n=7758)			PSM data set(n=6447)		
	CRT+IMT	CRT	P value	CRT+IMT	CRT	P value
	n=1363	n=6395		n=1357	n=5090	
Sex:			1			0.784
Female	342 (25.1%)	1605 (25.1%)		342 (25.2%)	1262 (24.8%)	
Male	1021 (74.9%)	4790 (74.9%)		1015 (74.8%)	3828 (75.2%)	
Age:			0.101			0.951
<=75	889 (65.2%)	4321 (67.6%)		883 (65.1%)	3319 (65.2%)	
>75	474 (34.8%)	2074 (32.4%)		474 (34.9%)	1771 (34.8%)	
Race:			<0.001			0.425
White	1125 (82.5%)	5150 (80.5%)		1125 (82.9%)	4247 (83.4%)	
Black	124 (9.10%)	854 (13.4%)		124 (9.14%)	488 (9.59%)	
Others	114 (8.36%)	391 (6.11%)		108 (7.96%)	355 (6.97%)	
Marital:			0.962			0.855
Unmarried and others	625 (45.9%)	2925 (45.7%)		624 (46.0%)	2324 (45.7%)	
Married	738 (54.1%)	3470 (54.3%)		733 (54.0%)	2766 (54.3%)	
Tumor location:			<0.001			0.491
Upper	220 (16.1%)	843 (13.2%)		217 (16.0%)	747 (14.7%)	
Middle	284 (20.8%)	1601 (25.0%)		284 (20.9%)	1171 (23.0%)	
Lower	743 (54.5%)	3340 (52.2%)		740 (54.5%)	2743 (53.9%)	
Overlapping	61 (4.48%)	262 (4.10%)		61 (4.50%)	222 (4.36%)	
Unknown	55 (4.04%)	349 (5.46%)		55 (4.05%)	207 (4.07%)	
Tumor size:			<0.001			0.395
<=60	704 (51.7%)	2908 (45.5%)		699 (51.5%)	2527 (49.6%)	
>60	213 (15.6%)	924 (14.4%)		212 (15.6%)	794 (15.6%)	
Unknown	446 (32.7%)	2563 (40.1%)		446 (32.9%)	1769 (34.8%)	
Grade:			0.004			0.082
Grade I-II	558 (40.9%)	2669 (41.7%)		558 (41.1%)	2132 (41.9%)	
Grade III-IV	486 (35.7%)	2475 (38.7%)		486 (35.8%)	1922 (37.8%)	
Unknown	319 (23.4%)	1251 (19.6%)		313 (23.1%)	1036 (20.4%)	
Histology:			<0.001			0.629
Adenocarcinoma	670 (49.2%)	2691 (42.1%)		666 (49.1%)	2471 (48.5%)	
Squamous cell carcinoma	556 (40.8%)	2822 (44.1%)		554 (40.8%)	2139 (42.0%)	
Others	137 (10.1%)	882 (13.8%)		137 (10.1%)	480 (9.43%)	
SEER historic stage:			0.575			0.514
Localized	419 (30.7%)	1914 (29.9%)		418 (30.8%)	1519 (29.8%)	
Regional	944 (69.3%)	4481 (70.1%)		939 (69.2%)	3571 (70.2%)	

### 3.2 Survival outcomes before and after propensity score matching

**Figure 4:**
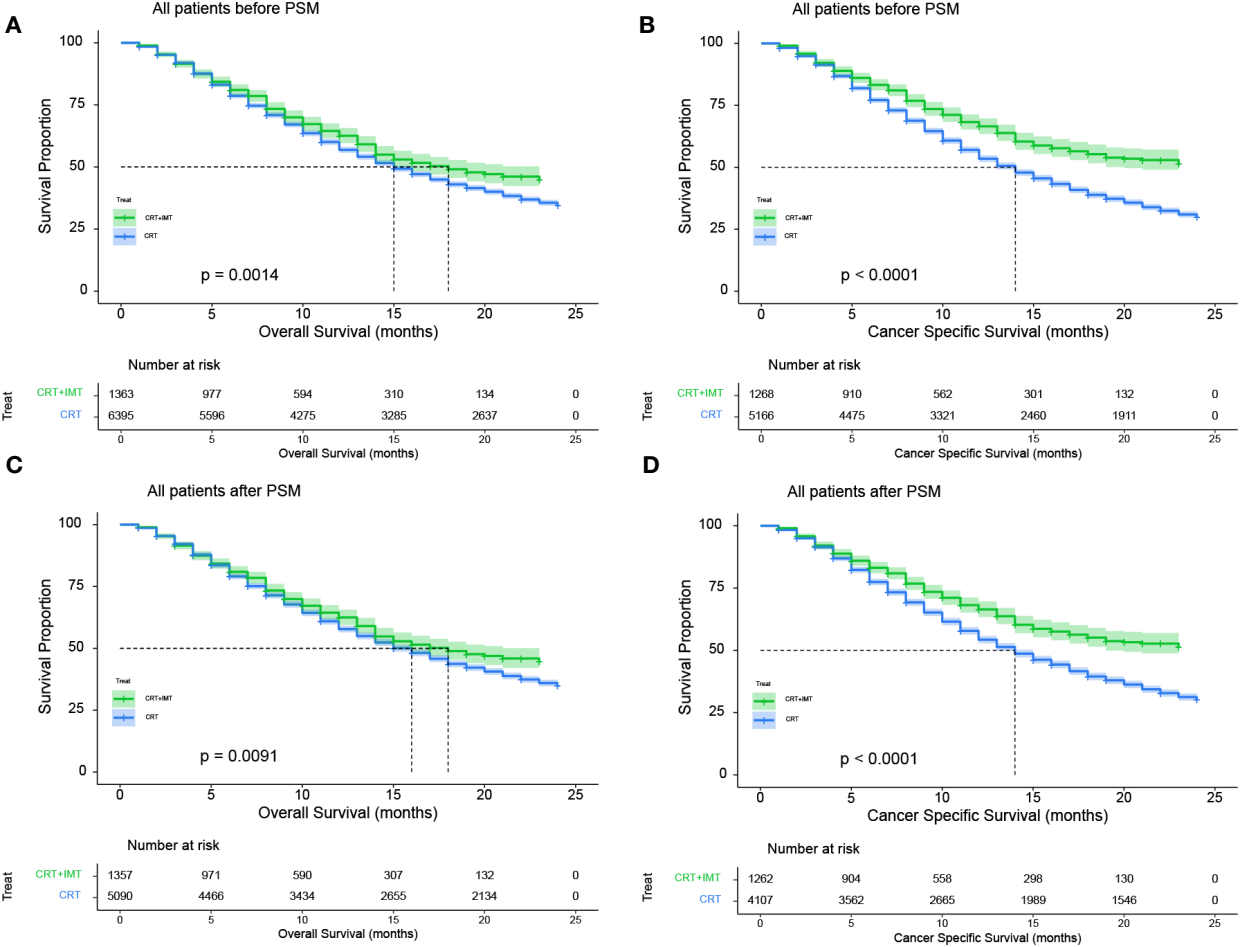
**(A–D)** Kaplan-Meier curves for OS **(A, C)** and CSS **(B, D)** of patients before and after propensity score matching. A 95% confidence interval (estimated from a log hazard), the number of patients at risk at different time points, and the P value for the log-rank test are displayed on the graph.

Before matching, the entire cohort had a median overall survival (OS) of 15 months (95% CI 15-16) and a median cancer-specific survival (CSS) of 14 months (95% CI 14-15). The ‘CRT+IMT’ group outperformed the ‘CRT’ group, with respective median OS values of 18 months (95% CI 15-23) and 15 months (95% CI 15-16). Notably, the median CSS for the ‘CRT+IMT’ group had not yet been reached (95% CI 20-NA), while the median CSS for the ‘CRT’ group was 14 months (95% CI 13-14). Remarkably, the results after propensity score matching exhibited no statistically significant disparities from those before matching. The entire cohort demonstrated a median OS of 16 months (95% CI 15-16) and a median CSS of 15 months (95% CI 14-15), whereas the ‘CRT+IMT’ group displayed a median OS of 18 months(95% CI 15-23) with an undetermined median CSS (95% CI 19-NA). Conversely, the CRT group featured a median OS of 16 months (95% CI 15-16) and a median CSS of 14 months(95% CI 13-15), as presented in **Table 2**. Kaplan-Meier curves corroborated these findings, indicating a significantly improved OS for patients in the CRT+IMT group compared to the CRT group (P=0.0014, **Figure 4A**). Similarly, the CSS for patients in the CRT+IMT group was markedly superior to that of the CRT group (P<0.001, **Figure 4B**). Post-propensity matching results indicated that chemoradiotherapy combined with immunotherapy yielded a noteworthy extension in both OS and CSS for patients (P=0.0091, **Figure 4C**; P<0.001, **Figure 4D**).

**Table 2:** Survival rates of patients stratified by treatment method.

Variables	Original data set(n=7758)				PSM data set(n=6447)			
	All patients	CRT+IMT	CRT	P value	All patients	CRT+IMT	CRT	P value
		n=1363	n=6395			n=1357	n=5090	
**Overall Survival**				P=0.0014				P=0.0091
6-months OS rate(95%CI)	79.0%(78.1%-79.9%)	81.0%(78.8%-83.3%)	78.6%(77.7%-79.7%)		79.4%(78.4%-80.4%)	80.9%(78.7%-83.2%)	79.1%(77.9%-80.2%)	
12-months OS rate(95%CI)	57.6%(56.5%-58.7%)	62.6%(59.6%-65.7%)	56.9%(55.7%-58.1%)		58.5%(57.2%-59.7%)	62.5%(59.5%-65.6%)	57.8%(56.4%-59.2%)	
18-months OS rate(95%CI)	43.6%(42.5%-44.8%)	49.1%(45.6%-52.8%)	43.0%(41.8%-44.2%)		44.3%(43.1%-45.6%)	48.9%(45.4%-52.7%)	43.7%(42.4%-45.1%)	
23-months OS rate(95%CI)	36.2%(35.1%-37.4%)	44.9%(40.6%-49.7%)	35.6%(34.4%-36.8%)		36.6%(35.4%-37.9%)	44.7%(40.3%-49.5%)	36.0%(34.7%-37.3%)	
Mean OS **(months)**	15 (15–16)	18 (15–23)	15 (15–16)		16 (15–16)	18 (15–23)	16 (15–16)	
**Cancer-specific Survival**				P<0.0001				P<0.0001
6-months CSS rate(95%CI)	78.1%(77.1%-79.2%)	83.2%(81.1%-85.5%)	77.1%(76.0%-78.3%)		78.6%(77.5%-79.7%)	83.1%(80.9%-85.4%)	77.4%(76.2%-78.7%)	
12-months CSS rate(95%CI)	55.3%(54.1%-56.6%)	66.5%(63.5%-69.7%)	53.5%(52.1%-54.8%)		56.4%(55.0%-57.8%)	66.4%(63.4%-69.7%)	54.3%(52.8%-55.8%)	
18-months CSS rate(95%CI)	40.8%(39.5%-42.0%)	55.3%(51.7%-59.2%)	38.9%(37.6%-40.2%)		41.7%(40.3%-43.1%)	55.1%(51.5%-59.0%)	39.5%(38.0%-41.0%)	
23-months OS rate(95%CI)	32.7%(31.5%-34.0%)	51.5%(46.9%-56.5%)	31.0%(29.7%-32.3%)		33.3%(31.9%-34.7%)	51.3%(46.7%-56.4%)	31.3%(29.9%-32.7%)	
Mean CSS **(months)**	14 (14–15)	Unreached(20-NA)	14 (13–14)		15 (14–15)	Unreached(19-NA)	14 (13–15)	

### 3.3 Cox regression analysis of survival

**Figure 5:**
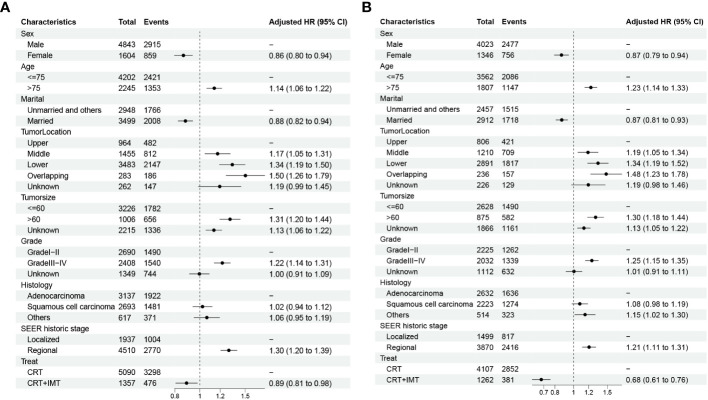
**(A, B)** Forest plots displaying the multivariable analysis of prognostic factors for OS **(A)** and CSS **(B)** in matched cohorts.

We conducted both univariable and multivariable Cox regression analyses for cancer-specific survival (CSS) and overall survival (OS) using the propensity score-matched data, as outlined in **Tables 3**, **4**. The univariable results revealed that sex, age, marital status, tumor location, tumor size, pathological grading, pathology type, SEER historic staging, and treatment modality exhibited significant associations with patients’ prognoses (P < 0.05). Characteristics with P-values < 0.1 in the univariable Cox regression analysis were subsequently included in the multivariable Cox regression analysis. The multivariable analysis indicated that sex, age, marital status, tumor location, tumor size, pathological grading, SEER historic staging, and treatment modality served as independent prognostic factors for both CSS and OS. Among these factors, being female, married, and receiving chemoradiotherapy combined with immunotherapy were associated with a more favorable prognosis, while age over 75 years, tumor size exceeding 60 mm, Grade III-IV tumors, and regional SEER historic staging were identified as risk factors. In contrast to the CRT group, the CRT+IMT group exhibited a pronounced survival advantage, resulting in a noteworthy 32% reduction in the risk for CSS (HR=0.68, 95% CI 0.61-0.76). For OS, the risk was reduced by 11% (HR=0.89, 95% CI 0.81-0.98), as visualized in **Figure 5**. Thus, the protective influence of chemoradiotherapy combined with immunotherapy on both CSS and OS was unequivocally established.

**Table 3:** Univariable and multivariable Cox regression analyses for overall survival of patients after propensity score matching.

Variables	N	Event N	Univariable			Multivariable		
			HR^1^	95% CI^1^	p-value	HR^1^	95% CI^1^	p-value
Sex								
Male	4,843	2,915	—	—		—	—	
Female	1,604	859	0.83	0.77, 0.89	<0.001	0.86	0.80, 0.94	<0.001
Age								
<=75	4,202	2,421	—	—		—	—	
>75	2,245	1,353	1.1	1.03, 1.17	0.005	1.14	1.06, 1.22	<0.001
Race								
White	5,372	3,147	—	—				
Black	612	377	1.07	0.96, 1.19	0.24			
Others	463	250	0.95	0.84, 1.08	0.446			
Marital								
Unmarried and others	2,948	1,766	—	—		—	—	
Married	3,499	2,008	0.93	0.88, 1.00	0.037	0.88	0.82, 0.94	<0.001
TumorLocation								
Upper	964	482	—	—		—	—	
Middle	1,455	812	1.19	1.06, 1.33	0.003	1.17	1.05, 1.31	0.006
Lower	3,483	2,147	1.4	1.27, 1.55	<0.001	1.34	1.19, 1.50	<0.001
Overlapping	283	186	1.61	1.36, 1.91	<0.001	1.5	1.26, 1.79	<0.001
Unknown	262	147	1.24	1.03, 1.49	0.021	1.19	0.99, 1.45	0.067
Tumor size								
<=60	3,226	1,782	—	—		—	—	
>60	1,006	656	1.4	1.28, 1.53	<0.001	1.31	1.20, 1.44	<0.001
Unknown	2,215	1,336	1.16	1.08, 1.24	<0.001	1.13	1.06, 1.22	<0.001
Grade								
GradeI-II	2,690	1,490	—	—		—	—	
GradeIII-IV	2,408	1,540	1.28	1.19, 1.37	<0.001	1.22	1.14, 1.31	<0.001
Unknown	1,349	744	1.01	0.92, 1.10	0.905	1	0.91, 1.09	0.932
Histology								
Adenocarcinoma	3,137	1,922	—	—		—	—	
Squamous cell carcinoma	2,693	1,481	0.86	0.81, 0.92	<0.001	1.02	0.94, 1.12	0.589
Others	617	371	1.01	0.90, 1.13	0.88	1.06	0.95, 1.19	0.285
SEER historic stage								
Localized	1,937	1,004	—	—		—	—	
Regional	4,510	2,770	1.32	1.22, 1.41	<0.001	1.3	1.20, 1.39	<0.001
Treat								
CRT	5,090	3,298	—	—		—	—	
CRT+IMT	1,357	476	0.88	0.80, 0.97	0.009	0.89	0.81, 0.98	0.017

–, the reference group.^1^HR, Hazard Ratio; CI, Confidence Interval.

**Table 4:** Univariable and multivariable Cox regression analyses for cancer-specific survival of patients after propensity score matching.

Variables	N	Event N	Univariable			Multivariable		
			HR^1^	95% CI^1^	p-value	HR^1^	95% CI^1^	p-value
Sex								
Male	4,023	2,477	—	—		—	—	
Female	1,346	756	0.86	0.79, 0.93	<0.001	0.87	0.79, 0.94	0.001
Age								
<=75	3,562	2,086	—	—		—	—	
>75	1,807	1,147	1.19	1.11, 1.28	<0.001	1.23	1.14, 1.33	<0.001
Race								
White	4,456	2,686	—	—				
Black	509	324	1.07	0.95, 1.20	0.253			
Others	404	223	0.94	0.82, 1.07	0.341			
Marital								
Unmarried and others	2,457	1,515	—	—		—	—	
Married	2,912	1,718	0.93	0.87, 1.00	0.036	0.87	0.81, 0.93	<0.001
TumorLocation								
Upper	806	421	—	—		—	—	
Middle	1,210	709	1.21	1.07, 1.36	0.002	1.19	1.05, 1.34	0.005
Lower	2,891	1,817	1.37	1.23, 1.53	<0.001	1.34	1.19, 1.52	<0.001
Overlapping	236	157	1.54	1.28, 1.85	<0.001	1.48	1.23, 1.78	<0.001
Unknown	226	129	1.21	0.99, 1.48	0.056	1.19	0.98, 1.46	0.086
Tumor size								
<=60	2,628	1,490	—	—		—	—	
>60	875	582	1.37	1.25, 1.51	<0.001	1.3	1.18, 1.44	<0.001
Unknown	1,866	1,161	1.16	1.07, 1.25	<0.001	1.13	1.05, 1.22	0.002
Grade								
GradeI-II	2,225	1,262	—	—		—	—	
GradeIII-IV	2,032	1,339	1.3	1.21, 1.41	<0.001	1.25	1.15, 1.35	<0.001
Unknown	1,112	632	1.01	0.92, 1.12	0.762	1.01	0.91, 1.11	0.909
Histology								
Adenocarcinoma	2,632	1,636	—	—		—	—	
Squamous cell carcinoma	2,223	1,274	0.9	0.84, 0.97	0.006	1.08	0.98, 1.19	0.118
Others	514	323	1.07	0.95, 1.20	0.275	1.15	1.02, 1.30	0.027
SEER historic stage								
Localized	1,499	817	—	—		—	—	
Regional	3,870	2,416	1.22	1.13, 1.32	<0.001	1.21	1.11, 1.31	<0.001
Treat								
CRT	4,107	2,852	—	—		—	—	
CRT+IMT	1,262	381	0.67	0.60, 0.74	<0.001	0.68	0.61, 0.76	<0.001

–, the reference group.^1^HR, Hazard Ratio; CI, Confidence Interval.

### 3.4 Subgroup analysis after PSM

**Figure 6:**
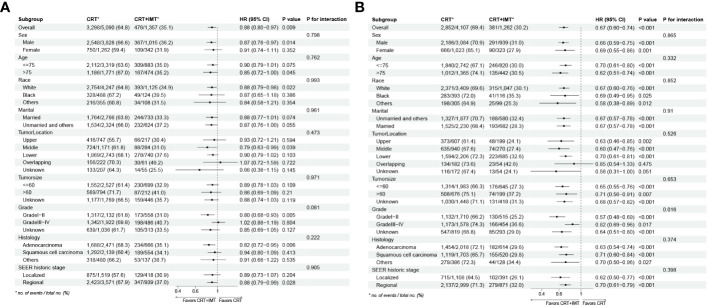
**(A, B)** Results for the subgroup analyses and interaction tests of treatment-based effects on OS **(A)** and CSS **(B)** are summarized in a Forest plot.

To further substantiate the impact of chemoradiotherapy combined with immunotherapy on the survival of patients afflicted with locally advanced unresectable esophageal cancer, the outcomes of subgroup analyses and interaction assessments, as depicted in **Figure 6**, indicated that the effect of this combined therapy on overall OS showed no significant difference across all nine subgroups (with all P-values for interaction >0.05). Similarly, the effect on CSS demonstrated no significant difference in eight subgroups (with P-values for interaction >0.05). However, an exception was noted in the Grade grading subgroups, where patients with grades 1-2 (HR=0.57,95%CI 0.48-0.69, P<0.001) experienced a more substantial survival advantage from chemoradiotherapy combined with immunotherapy compared to those with grades 3-4 (HR=0.82,95%CI 0.69-0.92, P=0.017; P for interaction <0.05).

### 3.5 Clinical and pathological data of our cohort

**Figure 7:**
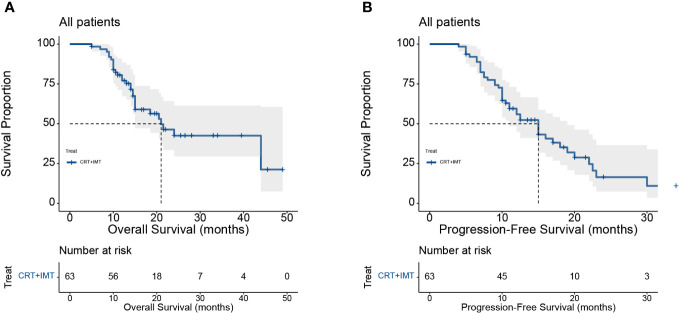
**(A, B)** Kaplan-Meier curves for OS **(A)** and PFS **(B)** of included patients in our institution.

Our cohort consisted of 63 patients diagnosed with locally advanced unresectable esophageal cancer. The baseline characteristics of the patients included in the analysis are presented in **Table 5**. All patients underwent combined chemoradiotherapy and immunotherapy. The median age of the patients in our study was 64 years, with an age range spanning from 44 to 83 years. All patients had squamous carcinoma as the pathologic type, and the median length of esophageal cancer lesions was 8 cm, ranging from 3.5 to 16 cm. First-line chemotherapy regimens were based on a combination of platinum and fluorouracil. The survival analysis is depicted in **Figure 7**, with a minimum follow-up period of 5 months, a maximum follow-up period of 49 months as of the last follow-up in August 2023, and a median follow-up duration of 20 months for all patients from the time of study inclusion to the last follow-up. The median overall survival (mOS) was 21 months (95% CI: 15 to not reached), and the median progression-free survival (PFS) was 15 months (95% CI: 11 to 19). The OS rates at 12, 18, and 24 months were 77.1%, 59.0%, and 42.5%, respectively. For PFS, the rates at 12, 18, and 24 months were 55.9%, 35.2%, and 16.5%, respectively. Among the 63 included patients, two patients achieved a complete response (CR), 37 patients attained a partial response (PR), 19 patients maintained stable disease (SD), and five patients experienced disease progression (PD). The overall response rate (ORR) for local relief was 62%, and the disease control rate (DCR) reached 92%.

**Table 5:** Baseline characteristics of included patients in our institution.

Variables	N = 63
Sex	
Male	56 (89%)
Female	7 (11%)
Age	
Median (IQR)	64.0(44- 83)
ECOG	
0	21 (33%)
1	42 (67%)
Grade	
G1	12 (19%)
G2	27 (43%)
G3	24 (38%)
TumorLocation	
Upper	31 (49%)
Middle	18 (29%)
Lower	14 (22%)
Tumor size	
Median (IQR)	8(3.5-16)
Tstage	
T1	3 (5%)
T2	12 (19%)
T3	30 (48%)
T4	18 (29%)
Nstage	
N0	5 (8%)
N1	45 (71%)
N2	12 (19%)
N3	1 (2%)
AJCC stage	
II	12 (19%)
III	33 (52%)
IVa	18 (29%)

## 4 Discussion

Immunotherapy has demonstrated remarkable efficacy across a spectrum of cancer types, including esophageal cancer (10–12). Our study contributes additional evidence to the realm of esophageal cancer, further bolstering the case for the effectiveness of combining chemoradiotherapy with immunotherapy, particularly for the specific patient population suffering from locally advanced unresectable esophageal cancer. The emergence of immunotherapy has ushered in a transformative era in the therapeutic field of esophageal cancer. Pivotal studies like KEYNOTE-181, ATRACTION-3, ESCORT, ORIENT-2 (phase II), RATIONALE302, and others have unequivocally established that, in cases where first-line chemotherapy proves ineffective in esophageal cancer, monotherapy with PD-1 inhibitors substantially enhances the median overall survival of patients. This has established the position of immunotherapy in the second-line treatment of advanced esophageal carcinoma. Our study delved into the SEER database to scrutinize the impact of combining immunotherapy with chemoradiotherapy on the prognosis of patients grappling with locally advanced inoperable esophageal carcinoma. The findings unequivocally demonstrated a substantial survival advantage among patients who underwent chemoradiotherapy in tandem with immunotherapy. This discovery offers invaluable insights into the realm of esophageal cancer treatment, particularly for individuals whose disease has advanced to the locally advanced and inoperable stages.

Our study encompassed a cohort of 7,758 patients diagnosed with locally advanced unresectable carcinoma of the esophagus, with diagnoses spanning the period from 2000 to 2020, as documented in the SEER database. As far as we are aware, this represents the first comprehensive data analysis of chemoradiotherapy combined with immunotherapy for locally advanced esophageal carcinoma. In 2018, the ASTRO conference reported the findings of a phase II trial that analyzed the effectiveness and safety of immunotherapy in combination with chemoradiotherapy for treating locally advanced esophageal squamous cell carcinoma (ESCC). Among the 16 enrolled patients, 14 were assessable for treatment efficacy. The results were promising, with one patient achieving a complete response (CR) at a rate of 7.1% and 13 patients (92.9%) showing complete or partial responses (13). Another phase Ib study discovered that the survival rate of locally advanced esophageal cancer patients receiving concurrent chemoradiotherapy along with immunotherapy was higher than previously reported for concurrent chemoradiotherapy without immunotherapy, marking a significant advancement in the use of immunotherapy in the context of locally advanced esophageal cancer (9). Due to the limitations of the SEER database in lacking relevant efficacy evaluation metrics, we were unable to assess patient responses or progression. Nevertheless, within our own institution, among the 63 patients who received combined chemoradiotherapy and immunotherapy, we achieved a concordant outcome with an overall response rate (ORR) of 62% and a disease control rate (DCR) of 92%. This aligns with the clinical trial results from prior studies involving partial chemoradiotherapy combined with immunotherapy, demonstrating favorable treatment efficacy and disease control among our patients. In the 2022 ASTRO conference, a retrospective clinical study was presented. In this study, 62 patients received induction immunotherapy in combination with chemotherapy, while 75 patients did not undergo induction therapy. The results indicated that the 1-year PFS rates were 72.6% and 60% (p=0.128), and the 1-year OS rates were 85% and 81.3% (p=0.058), respectively. This study suggested that, compared to concurrent chemoradiotherapy, immunotherapy combined with chemotherapy induction treatment followed by concurrent chemoradiotherapy results in better survival outcomes for locally advanced inoperable esophageal squamous cell cancer. Our research findings are consistent with previous research, demonstrating that the survival period of the group receiving chemoradiotherapy combined with immunotherapy is markedly superior to that of the chemoradiotherapy-only group. Furthermore, the 1-year OS rate for patients in our cohort who received chemoradiotherapy combined with immunotherapy was 77.9%. This is notably advantageous compared to some prior curative chemoradiotherapy trials. However, it is interesting to note that the 1-year OS rate for patients sourced from the SEER database who received chemoradiotherapy combined with immunotherapy was 62.5%, which is lower than in our own research cohort and some clinical trials. The discrepancy in these results may be attributed to substantial differences in the distribution of esophageal cancer pathological types between the United States and China. Adenocarcinoma is the predominant pathological type of esophageal cancer in the United States, while squamous cell carcinoma is predominant in China (1). Studies have suggested that patients with esophageal adenocarcinoma who receive chemoradiotherapy have poorer overall survival and tumor-specific survival in comparison to those with squamous cell carcinoma. This variation in chemoradiotherapy response could be attributed to differences in the characteristics of adenocarcinoma and squamous cell carcinoma (14). Furthermore, it might be related to the early diagnosis of esophageal cancer in China, which is based on endoscopic screening, while the United States lacks such screening, potentially resulting in more advanced stages at diagnosis (15).

While the mechanism of action for the combination of chemoradiotherapy and immunotherapy was not directly investigated in this study, several possible explanations can be considered based on previous research. First, chemoradiotherapy may induce the release of tumor neoantigens by potentially triggering immunogenic cell death (ICD) in cancer cells. ICD initiates pathways that facilitate the immune system’s recognition of dying cancer cells, playing a pivotal role in immunotherapy (16, 17). The immune system relies on identifying these antigens to target and attack the tumor. Second, chemoradiotherapy can reduce tumor burden and shrink tumor volume. Smaller tumor burden and volume make it easier for the immune system to detect and eliminate tumor cells, thus enhancing the effectiveness of immunotherapy (18). This effect may be particularly significant during the early stages of immunotherapy when the tumor has not yet developed mechanisms to evade immune surveillance. Third, immunotherapy may help enhance the abscopal effect during radiotherapy and overcome radiotherapy resistance. The abscopal effect relies on the presence of T cells, and radiotherapy may induce T cell depletion by increasing PD-L1 expression through the cGAS-Sting pathway, contributing to immune escape, reducing the abscopal effect, and increasing resistance to radiotherapy (19, 20). Blocking the PD-L1 receptor can reverse T cell depletion, maintaining T cell immune homeostasis, which, in turn, enhances the abscopal effect during radiotherapy and overcomes radiotherapy resistance (21, 22). Furthermore, tumor resistance to radiotherapy is often linked to tumor hypoxia caused by vascular abnormalities or dysfunction. Immune checkpoint inhibitors have been shown to normalize tumor vasculature, reduce tumor hypoxia, and increase tumor sensitivity to radiotherapy. This normalization of the tumor microenvironment can enhance the therapeutic effects of radiotherapy in combination with immunotherapy (23).

He et al. analyzed data from the SEER database and found a higher incidence of esophageal cancer in males when contrasted with females, displaying a male-to-female ratio of 2.91:1. Despite the higher incidence in males, females had better five-year survival rates (24). Davidson M et al. conducted a study pooling data from patients with esophageal and gastric cancers (25). They reported that females exhibited notably improved overall survival and progression-free survival compared to males. In our research, the male-to-female ratio for esophageal cancer patients was 2.98:1. Cox multifactorial regression analysis in our study revealed that females had significantly better OS and CSS compared to males. These findings from the investigations conducted by Hai et al. and Michael et al., as well as the results of our research, collectively emphasize the significant influence of gender on the occurrence and survival outcomes of esophageal carcinoma. Understanding the impact of gender on prognosis can aid in tailoring treatment strategies and improving the overall care of patients with this challenging disease.

Tumor size typically offers insights into the tumor’s growth and infiltration, whereas larger tumors signify deeper infiltration and later-stage classification. It has been suggested that the extent of lymph node metastasis is also a pivotal determinant affecting the prognosis of esophageal cancer, with tumor size identified as a high-risk factor for lymph node metastasis. Consequently, patients with larger tumors tend to exhibit a poorer prognosis. Numerous studies have unveiled the correlation between tumor length and the prognosis of esophageal cancer, aligning with the outcomes of our research (26, 27). This study has ascertained that tumor size stands as a significant predictor for patients afflicted with unresectable locally advanced esophageal cancer. The larger the tumor, the shorter its anticipated lifespan.

In this investigation, age underwent categorization into two distinct groups facilitated by the X-tile software. The univariable and multivariable COX analysis of the SEER cohort unequivocally demonstrated age to be an autonomous determinant of patients’ prognoses. Furthermore, it unveiled that the older the age, the graver the patient’s prognosis. The median age at which esophageal cancer is diagnosed typically hovers around 65 years, and with the gradual aging of the population, there is a noticeable increase in the proportion of elderly patients (28). The burgeoning adult population size and the process of population aging are the predominant factors contributing to the escalating number of cancer-related fatalities (1). Previous research conducted by Qiu et al. corroborated these findings, as they established that the overall survival of patients aged over 70 years diagnosed with stage I-II esophageal cancer was significantly shorter when contrasted with patients under the age of 70 (29). This underlines the close interconnection between the age of esophageal cancer patients and their overall prognosis, further validating the outcomes of this current study.

The prognosis of cancer patients is intricately linked not only to tumor size, pathological type, tumor staging, and treatment modalities employed but also to the psychosocial aspects of the patients. Recent years have witnessed a growing exploration of psychosocial factors in the context of malignant tumors. Marital status, among these critical psychosocial factors, has been substantiated to exert a pronounced influence on the etiology and survival prospects of numerous cancers, esophageal cancer included (30–33). A meta-analysis conducted in 2023 showed that married patients had higher OS and CSS rates than those who were unmarried, regardless of the type of cancer (34). This observation finds support in the present study as well. Several factors underpin this phenomenon. First and foremost, unmarried patients may encounter comparatively reduced financial support compared to their married counterparts, resulting in diminished receptiveness to oncological treatments and poorer treatment adherence (35). This can be attributed to the second factor, which pertains to the greater likelihood of unhealthy behaviors like smoking and alcohol consumption among unmarried patients, ultimately contributing to less favorable prognoses (36). Lastly, the absence of support from spouses or family members places unmarried patients at a heightened risk of developing psychological conditions such as anxiety and depression, which can further compound their challenges on the road to recovery (37, 38).

It is essential to recognize the constraints of this study, even though it has provided helpful information. First, the SEER database lacks detailed information regarding chemoradiotherapy and immunotherapy. Second, the relatively short follow-up duration for patients in the immunotherapy era of esophageal cancer within the SEER database hinders the assessment of long-term survival outcomes. Third, the SEER database offers limited prognostic data, encompassing only OS and CSS, precluding the analysis of objective response rates (ORR), disease-control rates (DCR), progression-free survival, and quality of life—significant metrics for evaluating treatment efficacy. Fourth, propensity score-matched analyses may not be able to accurately account for unmeasured confounding variables between groups, which could lead to an inaccurate outcome.

Furthermore, the results of this research point toward promising directions for future research. Firstly, additional clinical trials could be initiated to corroborate the efficacy of chemoradiotherapy combined with immunotherapy in locally advanced unresectable esophageal cancer. Secondly, investigations into the impacts of varying combinations of immunotherapeutic agents with chemotherapeutic regimens, the timing and dosages of radiotherapy, and the optimal treatment duration on the prognosis of locally advanced inoperable esophageal carcinoma could be pursued to ascertain the most suitable treatment regimen for patients. Lastly, an individualized approach to treatment should be incorporated into the strategy for locally advanced inoperable esophageal carcinoma. Given the potential variability in the effects of immunotherapy among different patients, identifying which individuals are most likely to benefit from this treatment will enable a more tailored treatment plan.

## 5 Conclusions

Considering the grim outlook for individuals with locally advanced unresectable esophageal cancer and the limited body of research regarding immunotherapy in this patient group, we undertook an examination of patients diagnosed with locally advanced unresectable esophageal cancer between 2000 and 2020, utilizing the U.S. SEER database. Our investigation demonstrated that patients who underwent chemoradiotherapy combined with immunotherapy experienced a substantial improvement in survival compared to those who received chemoradiotherapy alone. Sex, age, marital status, tumor location, tumor size, pathologic grade, SEER historic staging, and treatment modality were all found to be independent prognostic factors for both OS and CSS in patients. In summary, our study contributes additional support to the field of esophageal cancer by confirming the effectiveness of chemoradiotherapy combined with immunotherapy in the specific patient subset of locally advanced unresectable esophageal cancer. This finding should be further substantiated through larger-scale trials.

## Data availability statement

The raw data supporting the conclusions of this article will be made available by the authors, without undue reservation.

## Ethics statement

Ethical approval was not required for the study involving humans in accordance with the local legislation and institutional requirements. Written informed consent to participate in this study was not required from the participants or the participants’ legal guardians/next of kin in accordance with the national legislation and the institutional requirements.

## Author contributions

LX: Methodology, Software, Visualization, Writing – original draft, Writing – review & editing. ZZ: Supervision, Writing – review & editing.
